# Attitudes Toward Cannabis Use During Labor in the United States

**DOI:** 10.1089/whr.2021.0125

**Published:** 2022-01-31

**Authors:** Brooke A. Chernek, Kara R. Skelton

**Affiliations:** Department of Health Sciences, College of Health Professions, Towson, Maryland, USA.

**Keywords:** marijuana, prenatal, pregnancy, childbirth

## Abstract

***Objective:*** Little is known about women's attitudes toward cannabis use during labor. We aim to address this gap by (1) reporting on attitudes toward cannabis use during labor, including cannabis use during most recent childbirth; and (2) examining the extent to which attitudes, willingness to use cannabis during labor, and cannabis use during most recent childbirth vary across state-level cannabis policies.

***Methods:*** In Spring 2021, we recruited biological women of reproductive age (18–40 years) for an online survey. We collected sociodemographic information and asked women about their attitudes toward cannabis use during labor, willingness to use cannabis during labor, and prior cannabis use during most recent childbirth. We ran descriptive statistics and used Fisher's exact tests to examine the association between state cannabis policies and attitudes toward cannabis use during labor, including willingness to use cannabis during labor.

***Results:*** In our sample (*N* = 163), most women reported they would either consider using (47.85%) or previously used (3.07%) cannabis during labor. Compared with women who would not use, women who reported willingness to use or prior use of cannabis during labor were more likely to report a lower annual household income (*p* = 0.001) and education level (*p* < 0.001). Women willing to consider cannabis use were also more likely to report prenatal cannabis use (*p* < 0.001) and reside in a state with recreational cannabis legalization (*p* = 0.003). Women who would not consider using cannabis during labor were more likely to perceive one or more risks of use compared with women who would consider using cannabis during labor (90.00% vs. 72.29%, respectively; *p* = 0.005). In fully illegal states, 66% of women reported they would be more likely to use cannabis during labor if it were legal.

***Conclusions:*** Future research is urgently needed to guide clinical practice. To mitigate adverse health outcomes, prenatal care providers should discuss cannabis use during labor with their patients.

## Introduction

Prenatal cannabis use is becoming increasingly prevalent in the U.S.^1,2^ Increasing prenatal cannabis use can be attributed to increasing social acceptance of use, decreasing risk perception, and cannabis policies that allow for recreational use and subsequent commercialization.^3–6^ A recent study of prenatal cannabis use in 14 states found prenatal cannabis use was more prevalent in states where recreational cannabis is legal, compared with states where recreational cannabis was illegal (6.29%, vs. 5.22%, respectively).^7^ However, increases in prenatal cannabis use,^1,2^ in combination increased mean potency of 9-carboxy-Δ9-tetrahydrocannabinol (THC), the main psychoactive ingredient in cannabis, elicits concern that associated adverse health effects of use may be much greater than what has been previously reported.^8,9^

Growing evidence supports there are numerous adverse maternal and neonatal risks associated with perinatal cannabis use. Prenatal cannabis use has been associated with adverse birth outcomes, such as preterm birth, low birth weight, and admission to the neonatal intensive care unit.^10^ In response to these potential risks, national maternal and child health organizations, including the American College of Obstetricians and Gynecologists (ACOG) and the American Academy of Pediatrics (AAP) recommend pregnant women recommend abstain from cannabis use.^9,11^ Additionally, as THC is lipophilic and is detectable in breast milk for up to 6 days after use,^12^ both ACOG, the AAP, and the Academy for Breastfeeding Medicine recommend that women abstain from using cannabis while breastfeeding.^11,13^

Prior evidence has found that the top cited reasons for prenatal cannabis use include to alleviate or reduce stress and anxiety, to relieve pain, and as an antiemetic.^14–16^ Undoubtedly, childbirth is a critical life event that is often associated with stress and anxiety, particularly surrounding labor pains, potential medical interventions, and health of mother and baby. Yet, few studies have examined cannabis use during labor and childbirth.^17,18^ A recent descriptive study examined willingness to use cannabis during labor among Canadian women and found that one-third of women would consider using cannabis for labor pain.^18^ However, this study was conducted before legalization of recreational cannabis in Canada in October 2018 and included women with vaginal deliveries only.^18^ Thus, this study's findings cannot be generalized to U.S. women and is even less applicable to U.S. women residing in states where recreational cannabis is legal.

There is an additional U.S.-based study that examined cannabis use for gynecological conditions and asked about use during labor.^17^ However, as this study was broadly focused on gynecological conditions, a detailed examination of willingness to use cannabis during labor was omitted.^17^ Thus, there has yet to be a focused examination of women's attitudes toward cannabis use during labor in the United States, including the extent to which attitudes toward cannabis use during labor and willingness to use vary across states with different cannabis policies.

The rapidly changing landscape of cannabis legality in the United States, in combination with increasing cannabis use among pregnant women, creates a need to examine attitudes toward cannabis use during labor. Currently, there are large evidence gaps on attitudes toward cannabis use during labor–a topic of both clinical and public health importance. To address this evidence gap, we aimed to examine attitudes toward cannabis during labor, including willingness to use and prior use of cannabis during labor, among women of reproductive age in the United States. Our secondary aim was to examine the extent to which willingness to use cannabis during labor varied across state-level cannabis policies.

## Materials and Methods

### Data source

This study presents a subset of results from a study examining cannabis-related knowledge, attitudes, influences, and use among U.S. women of reproductive age. We conducted a cross-sectional survey in April of 2021. In March 2021, we recruited biological women of reproductive age for an online survey. We used a purposeful sampling strategy to recruit a geographically diverse sample of women. Specifically, we used the Facebook advertising platform to recruit participants, which enables us to effectively reach a large sample pool within our target population.^19,20^ In the Facebook advertisement, we asked women who were interested in participating the research study to message the study page. Interested women were then screened by a research assistant for the following inclusion criteria: (1) biological female, (2) between the ages of 18 and 40; and (3) current resident of the United States.

The use of participant screening by a research assistant served as a validation point for email addresses and helped to eliminate single-user repeated attempts on the survey. Upon verification of eligibility criteria, we distributed a unique survey link to each participants' email using Qualtrics, an online survey platform.

The first section of the survey included an informed consent, in which participants selected, “Yes, I agree to participate in this study,” or “No, I do not agree to participate in this study.” Only those participants who agreed to participate could advance to the survey and the survey could not be completed more than once. Survey responses, including participants who did not agree to participate (*n* = 5), were automatically recorded in Qualtrics. Upon verified completion of the survey, participants received a $15 Amazon gift card sent to their email address.

The online survey was developed by the research team after performing a systematic scoping review of existing literature. We asked women about their cannabis-related knowledge, attitudes, and use (including prior history of use and current use). We also included specific items to assess willingness to use cannabis during labor and attitudes toward cannabis use during labor. After obtaining a Certificate of Confidentiality from the federal government, the Towson University Institutional Review Board approved this study.

### Measures

#### Maternal characteristics

We collected data on maternal sociodemographic characteristics in the survey. These characteristics included age, annual household income, race, marital status, and education level. We also asked women if they currently had a medical marijuana card and history of prenatal cannabis use. We characterized women's prenatal cannabis use into three categories: never users (reported never using cannabis during pregnancy), ever user (reported ever using cannabis during pregnancy, but stopped using at some point during pregnancy), and continued user (reported continued cannabis use throughout entire pregnancy).

#### Willingness to use cannabis during labor

The following question was asked to assess willingness to use cannabis during labor: “Please select which of the following best describes your attitude toward using cannabis during labor and childbirth?” Response options were, “I would not consider using cannabis during labor,” “I would consider using cannabis during labor,” and “I used cannabis during my most recent childbirth.”

#### Attitudes toward cannabis use during labor

The question, “Please rate the degree to which you agree or disagree with each of the following statements related to legalization of cannabis for adult use (recreational use),” was asked to assess how recreational cannabis legalization may impact willingness to use cannabis during labor. This was a Likert-scale question, in which responses ranged from strongly disagree to strongly agree; a separate response option, not applicable-cannabis is already legal in my state, was also available. For analysis of this variable, we excluded women residing in a legal state and recoded remaining responses into “disagree and agree,” creating a single dichotomous variable for attitudes toward cannabis use during labor.

#### Risks of cannabis use

We asked participants the following question, “What do you believe are the risks of using cannabis (select all that you believe are risks).” Response options included: addiction to cannabis, impaired memory, increased use of other drugs, legal problems, personal or relationship problems, decrease in intelligence, increase in stress, anxiety or depressions, sleep disruptions, new or worsening health problems, no risks, and other. For purposes of this study, we dichotomized responses into “one or more risks” and “no risk.”

#### State-level cannabis policies

We asked participants their current state of residence, with a drop list of all U.S. states and territories. Using state of residence and state-level legislation effective dates, we created a three-level indicator variable of state-level cannabis policies: fully legal (includes women residing in states with recreational and medicinal cannabis legalization as of April 2021), mixed (includes women residing in states with medicinal cannabis legalization or cannabis decriminalization as of April 2021), and fully illegal (includes women residing in states with neither recreational nor medicinal cannabis legalization or cannabis decriminalization as of April 2021).

### Statistical analyses

For all analyses, we used Fisher's exact test and a statistical significance level of alpha = 0.05. First, we compared sociodemographic characteristics between women who would consider using or previously used cannabis during labor and those who would not consider using cannabis during labor. Next, we examined willingness to use cannabis during labor by state-level cannabis policies (fully legal, mixed, fully illegal). We then examined cannabis benefit and risk perceptions by willingness to use cannabis during labor. In our final analysis, we examined attitude toward using cannabis during labor by state-level cannabis policies (mixed vs. fully illegal). We performed all data analyses using Stata 16.1 (College Station, TX).

## Results

Of the 172 women who consented to our survey, 9 were missing responses on attitudes toward cannabis use during labor and were excluded from analysis. Thus, the final analytic sample includes 163 women with complete data. Approximately 54.0% of our sample resided in a state with mixed cannabis policies, 38.04% resided in states where cannabis is fully legal, and 8.0% resided in a state where cannabis is fully illegal.

A majority of women were between the ages of 18 and 34 (68.71%), with a mean age of 29.92 (standard deviation = 6.61). Table 1 displays sociodemographic characteristics of the sample, stratified by willingness to use cannabis during labor. The sample was predominately low income, with 53.09% reporting an annual household income less than or equal to $39,999; most participants were White (77.16%). Most women reported being currently pregnant (45%) and 54.7% of women with children reported their child was 3 years of age or less, representing women with a recent childbirth.

**Table 1.** Sociodemographic Characteristics of Women by Views on Using Cannabis During Labor, ***N*** = 163

Characteristic	Total sample (***N*** = 163)	Would consider using cannabis^a^ (***n*** = 83)	Would not consider cannabis use (***n*** = 80)	***p*** ^b^
Age				0.260
18–24	43 (26.38)	26 (31.33)	17 (21.25)	
25–34	69 (42.33)	35 (42.17)	34 (42.50)	
35–40	51 (31.29)	22 (26.51)	29 (36.25)	
Income				0.001
≤$39,999	86 (53.09)	57 (68.67)	29 (36.71)	
$40,000–$69,999	31 (19.14)	12 (14.46)	19 (24.05)	
$70,000–$99,999	30 (18.52)	10 (12.05)	20 (25.32)	
≥$100,000	15 (9.26)	4 (4.82)	11 (13.92)	
Race				0.591
Black	9 (5.56)	3 (3.66)	6 (7.50)	
White	125 (77.16)	65 (79.27)	60 (75.00)	
Other race^c^	28 (17.26)	14 (17.07)	14 (17.50)	
Hispanic				0.566
Yes	18 (11.04)	9 (10.84)	9 (11.25)	
No	145 (88.96)	74 (89.16)	71 (88.75)	
Marital status				0.211
Married	77 (47.24)	35 (42.17)	42 (52.50)	
Unmarried	86 (52.76)	48 (57.83)	38 (47.50)	
Education				<0.001
High school or less	41 (25.15)	28 (33.73)	13 (16.25)	
Some college	38 (23.31)	26 (31.33)	12 (15.00)	
College degree	56 (34.36)	26 (31.33)	30 (37.50)	
Graduate degree	28 (17.18)	3 (3.61)	25 (31.25)	
Medical card				1.00
No	116 (89.23)	55 (47.41)	61 (89.71)	
Yes	14 (10.77)	7 (11.29)	7 (10.29)	
Prenatal cannabis use				<0.001
Never user	38 (39.58)	9 (20.93)	29 (54.72)	
Ever user	36 (37.50)	17 (39.53)	19 (35.85)	
Contined user	22 (22.92)	17 (39.53)	5 (9.43)	

^a^ Includes participants who reported they would consider using cannabis for labor pain (*n* = 78) as well as those who reported they previously used cannabis for labor pain (*n* = 5).

^b^ *p*-Value from Fisher's exact.

^c^ includes Asian or Asian American, Native Hawaiian or Other Pacific Islander, American Indian or Alaskan Native, or other races.

Compared with those who would not consider using cannabis during labor, women who would consider using or reported prior use of cannabis during labor were more likely to report a lower annual household income (*p* = 0.001) and education level (*p* < 0.001). Women who were willing to consider cannabis use were also more likely to report prenatal cannabis use during their current or most recent pregnancy (*p* < 0.001). No differences in age, race, or marital status were found.

Table 2 describes willingness to use cannabis during labor by state-level cannabis policies. Overall, a majority (50.92%) of women reported they would either consider using cannabis use for labor pains or previously used cannabis for labor pains during their most recent childbirth. Of the small number (3.07%) of women who reported using cannabis during their most recent labor, 80% resided in a state where recreational cannabis was legal. We found a significant association between willingness to use cannabis and recreational cannabis legalization (*p* = 0.033). Half (50.0%) of the women residing in states where recreational cannabis is legal reported they would not consider using cannabis.

**Table 2.** Willingness to Use Cannabis During Labor by State-Level Cannabis Policies, ***N*** = 163

Survey question	Total	Fully legal (***n*** = 62)^a^	Mixed (***n*** = 88)^b^	Fully illegal (***n*** = 13)^c^
Willingness to use cannabis during labor				
Would consider using cannabis	78 (47.85)	27 (43.55)	45 (51.14)	6 (45.15)
Would not consider using cannabis	80 (49.08)	31 (50.00)	42 (47.73)	7 (53.85)
Used cannabis during most recent childbirth	5 (3.07)	4 (6.45)	1 (1.14)	0 (0.00)

^a^ Includes women residing in states with recreational and medicinal cannabis legalization as of April 2021.

^b^ Includes women residing in states with medicinal cannabis legalization or cannabis decriminalization as of April 2021.

^c^ Includes women residing in states with neither recreational nor medicinal cannabis legalization or cannabis decriminalization as of April 2021.

Overall, most women reported that a benefit of cannabis use was pain management (93.25%) (Table 3). Table 3 reports participants' cannabis benefit and risk perceptions stratified by willingness to use cannabis during labor. Most participants (80.98%) also perceived one or more risks associated with cannabis use. Women who would not consider using cannabis during labor were more likely to perceive one or more risks associated with cannabis use compared with those women who would consider use (*p* = 0.005).

**Table 3.** Cannabis Benefit and Risk Perceptions by Willingness to Use Cannabis During Labor (***N*** = 163)

Survey question	Total sample	Would consider using cannabis (***n*** = 83)^a^	Would not consider using cannabis (***n*** = 80)	***p*** ^b^
Cannabis use benefit pain management				
Yes	152 (93.25)	79 (95.18)	73 (91.25)	0.364
No	11 (6.75)	4 (4.82)	7 (8.75)	
Risk of cannabis use				
One or more risks	132 (80.98)	60 (72.29)	72 (90.00)	0.005
No risk	31 (19.02)	23 (27.71)	8 (10.00)	

^a^ Includes participants who reported they would consider using cannabis for labor pain (*n* = 78) as well as those who reported they previously used cannabis for labor pain (*n* = 5).

^b^ *p*-Value from Fisher's exact.

Table 4 reports participants' attitudes toward cannabis use during labor by state-level cannabis policies. Among participants (63.53%) who resided in a state with mixed cannabis policies, most (63.53%) reported they would be more likely to use cannabis to relieve pain during labor and childbirth if it were legal in their state. Similarly, 66.67% of participants residing in a state where cannabis is fully illegal reported they would be more likely to use cannabis during labor if it were legal in their state.

**Table 4.** Attitudes on Cannabis Use During Labor by State-Level Cannabis Policies, ***N*** = 97

Survey question	Mixed (***n*** = 85)^a^	Fully illegal (***n*** = 12)^b^	***p***
I would be more likely to use cannabis to relieve pain during labor/childbirth if it were legal in my state			0.556
Disagree	31 (36.47)	4 (33.33)	
Agree	54 (63.53)	8 (66.67)	

Excludes participants residing in a state with legalized recreational cannabis (*n* = 27).

^a^ Includes women residing in states with medicinal cannabis legalization or cannabis decriminalization as of April 2021.

^b^ Includes women residing in states with neither recreational nor medicinal cannabis legalization or cannabis decriminalization as of April 2021.

## Discussion

In this cross-sectional study, we examined attitudes toward cannabis use during labor and willingness to use cannabis during labor in a geographically diverse sample of U.S. women of reproductive age. We also examined how attitudes toward cannabis use varied across state-level cannabis policies. Our findings have implications for prenatal care clinicians, researchers, and policymakers.

This is one of the first studies to examine cannabis use during labor in the United States. Postonogova et al. examined Canadian women's attitudes toward cannabis use during labor before legalization of cannabis in Canada and found one-third of women would consider the use of cannabis for labor pain, although many are unsure of its effects.^18^ Comparatively, we found that over half (50.92%) of women in our study would consider using cannabis during labor. This is a surprising finding, as there are no existing studies supporting neither the safety nor efficacy of cannabis use during labor.

There are existing studies examining health outcomes (both maternal and infant) associated with prenatal cannabis use.^21–24^ However, there is a paucity of research and evidence on the health effects of cannabis use during labor. One case study by Tuncali presents a case of persistent perioperative tachycardia in postoperative patient who underwent an emergency cesarean section under combined spinal epidural anesthesia.^25^ It was only after persistent postoperative tachycardia did the patient disclose cannabis use on the same day of the procedure (6 hours before admission), citing legal concerns as the reason for nondisclosure. Thus, there is some evidence that cannabis interacts with medications given during labor and is of clinical importance to examine.

The limited amount of research conducted in this area underscores the need for further research in this area to examine the pharmacokinetics of specific cannabis products (*e.g.*, concentrates, edibles, smoking) and their interaction, if any, with standard medications used during labor and delivery, including cesarean sections. Additionally, future research should examine effective screening strategies to increase and encourage cannabis disclosure upon admittance for labor and delivery.

Prior studies have found an increase in maternal cannabis use prevalence after recreational cannabis legalization,^26^ including prenatal cannabis use.^22^ We hypothesized that women residing in states where recreational cannabis is fully legal were more likely to report a willingness to use cannabis or prior use of cannabis during labor. Indeed, we found a significant association between willingness to use cannabis and recreational cannabis legalization (*p* = 0.033). Approximately 64% of women who resided in a state yet to legalize recreational cannabis reported they would be more likely to use cannabis during labor if it were legal in their state.

Our findings suggest that cannabis legalization may bring with it increases in cannabis use during labor—a point of concern given the lack of evidence in this area. Federally, cannabis use remains illegal in all states, making development of clinical recommendations on cannabis from a legal standpoint inherently complex.^13^ Nonetheless, clinical guidance from both state and national organizations is urgently needed.

Current guidance from the ACOG recommends that clinicians ask women about prenatal cannabis use for medical and nonmedical reasons, counsel women reporting about adverse health consequences of continued use during pregnancy, and encourage women to discontinue cannabis use.^11^ However, ACOG has not released labor-specific clinical guidance, presenting a large clinical gap. Given the paucity of evidence on the interaction between cannabis and medications used during labor and delivery, it is imperative that obstetricians and other prenatal care providers discuss cannabis use during labor in accordance with national guidance, which includes screening for cannabis use upon delivery.

Although 93.25% of women reported a benefit of cannabis use was pain management, we found that cannabis risk perceptions, but not benefit perceptions, were associated with willingness to use cannabis. Among women who would not consider cannabis use, 90.0% perceived one or more risk(s) of cannabis use, compared with only 72.29% of women who would consider using cannabis perceiving one or more risk(s) (*p* = 0.005). Thus, discussing risks of cannabis use during labor may be an important prevention strategy for future interventions aiming to reduce uptake of cannabis use during labor, although future research is needed to confirm this finding.

Future studies should aim to examine what antecedents of cannabis use, such as risk perceptions, knowledge, and beliefs are associated with use of cannabis during labor and willingness to use cannabis during labor. Ultimately, there is also a need to examine what sociodemographic factors, in addition to the ones identified in this study, may be associated with willingness to use cannabis using a larger, more racially diverse sample.

## Limitations

The results of our study should be interpreted in the context of a few limitations. First, we had a relatively small sample size (*N* = 163), which was intentional as we aimed to examine psychometric properties. Second, as attitudes toward cannabis during labor have not yet been examined in the United States, this study was primarily descriptive in nature. Future studies should aim to address this limitation through inferential analyses with a larger sample of women. Likely, prior use of cannabis would be a predictor of cannabis use during labor. Future studies should aim to examine predictors of cannabis use during labor.

Social desirability may have impacted participants' responses on this survey. However, we aimed to increase accurate disclosure through protection of confidentiality and use of nonstigmatizing language on our survey. Similarly, it is also possible that women in states with legalized recreational cannabis felt more comfortable disclosing preconception and postpartum cannabis use. Lastly, this is a cross-sectional survey and causality cannot be determined; future research should aim to utilize a time series design to establish temporality.

## Conclusion

In this cross-sectional study, we found nearly half (47.08%) of women reported they would consider using cannabis during labor and that women. Our findings also suggest that cannabis legalization may bring with it increases in cannabis use during labor. Future research is urgently needed in this area to examine not only cannabis use patterns of women during labor, but also potential interactions between cannabis and other drugs commonly used during labor and delivery. This research is urgently needed to guide clinical practice and protect against potential adverse health outcomes associated with cannabis use during labor.

## Abbreviations Used

AAP: American Academy of Pediatrics
ACOG: American College of Obstetricians and Gynecologists
THC: 9-carboxy-Δ9-tetrahydrocannabinol
